# Radiological outcome of bone disease in multiple myeloma patients pre- and post therapy: A single center experience

**DOI:** 10.15537/smj.2022.43.8.20220055

**Published:** 2022-08

**Authors:** Somaya A. Aljohani, Israa H. Saib, Ghulam Shah Syed, Ahmed Alruwaili, Shah P. Numani, Sulaiman A. Almuthri, Ramesh K. Vishwakarm, May Anne C. Mendoza, Mohammed J. Alsalman, Nabil M. Ozair, Ayman Y. Alhejazi, Ahmed S. Alaskar, Giamal E. Gmati

**Affiliations:** *From the Department of Internal Medicine (Aljohani, Alsalman, Ozair), King Abdulaziz Medical City Ministry of National Guard - Health Affairs, King Abdullah International Medical Research Center, King Saud Bin Abdulaziz University for Health Sciences; from the Department of Nuclear Medicine (Saib, Syed), King Abdulaziz Medical City Ministry of National Guard - Health Affairs, King Abdullah International Medical Research Center, King Saud Bin Abdulaziz University for Health Sciences; from the Division of Adult Hematology, Department of Oncology (Alhejazi, Alaskar, Gmati), King Abdulaziz Medical City Ministry of National Guard - Health Affairs, King Abdullah International Medical Research Center, King Saud Bin Abdulaziz University for Health Sciences; from the Department of Biostatistics (Vishwakarm), King Abdullah International Medical Research Center, King Saud Bin Abdulaziz University for Health Sciences; and from the Clinical Trial Services (Mendoza), King Abdullah International Medical Research Center, King Saud Bin Abdulaziz University for Health Sciences, Riyadh, Kingdom of Saudi Arabia.*

**Keywords:** multiple myeloma, PET scan, bone disease, outcome

## Abstract

**Objectives:**

To find any correlation between the clinical response as per International Working Myeloma Group (IWMG) response criteria and the radiological response at the end of treatment.

**Methods:**

A retrospective cohort study was conducted, total of 39 patients whom diagnosed with multiple myeloma (MM) between January 2010 and December 2018 and fulfilled the study criteria were included.

**Results:**

The high sensitivity and specificity of positron emission tomography/computed tomography (PET/CT) in detecting osteolytic myeloma lesions in the bones was strongly emphasized in our study. Follow up PET/CT, we found that while 17 patients showed complete remission in PET/CT, and 14 of these of patients demonstrated a complete clinical response at end of therapy assessment.

**Conclusion:**

Although we did not find a statistically significant correlation between the response versus metabolic activity and the number of bone/bone marrow lesions, however, our study was limited by the absence of clear criteria for defining disease response in PET/CT in MM patients. Further prospective analysis would be needed to establish a defined criterion.

**M**ultiple Myeloma (MM) is a malignant neoplastic proliferation of clonal plasma cells producing monoclonal immunoglobulins which infiltrate multiple organs. When disease progression of MM causes end-organ damage, it is often characterized by hypercalcemia, renal failure, and anemia and bone destruction in the form of osteopenia, osteolytic lesions, or pathological fractures. It is estimated that 24,050 new cases of myeloma were diagnosed in 2014, and 11,090 people died from the disease in the United States. In Saudi Arabia, it accounts for approximately 1% of all malignancies, and in 2018 there were 252 newly diagnosed cases.^1^ In previous decades, standard skeletal survey used to be the gold-standard initial modality to detect bone lytic lesions in MM patients, although it only detects lesion when 30%-50% of bone mass is destroyed.^2^ Modern imaging modalities are now being implemented for assessing myelomatous bony lesions, such as whole body-magnetic resonance imaging (WB-MRI), low-dose computed tomography (CT), and positron emission tomography (PET) with 2-deoxy-2-fluorine-18-fluoro-D-glucose (18FDG-PET). In a former systemic review comparing these different modalities to whole body skeletal radiography, it showed that the modern imaging techniques have detected up to 80% more lesions than conventional radiography, with CT and MRI being equally sensitive.^3^ Another study showed that there is no difference in the detection of bone lesions at the time of diagnosis between WB-MRI and 18-FDG-PET-CT.^4^ However, MRI is usually reserved for areas such as spinal lesions, while 18F-FDG PET/CT is for a more detailed osseous or extra-osseous whole-body involvement.^5^

Several studies have demonstrated the value of 18F-FDG PET/CT as part of the initial work-up for diagnosing plasmacytoma and MM. One prospective study, designed to quantify bone marrow infiltration and distribution pattern in F-FDG PET/CT, reported a higher tracer uptake with higher infiltration, whilst it showed a negative PET/CT with the lowest bone marrow infiltration.^6^ Due to low sensitivity of skeletal survey in monitoring response to therapy or detecting changes in the skeletal burden of the disease, the use for more advanced techniques is needed for more precise assessment of response to therapy and for prognostication purposes.^7^ Availability of advanced therapeutic options such as stem cell transplantation also require accurate assessment of the treatment.^8^ In the recent consensus statement article, the International Myeloma Working Group highlighted the effectivity of F-FDG PET/CT in detecting minimal residual disease (MRD) which remains a questionable tool that needs further evaluation, keeping in mind that false positive and false negative results can happen due to low sensitivity of PET/CT in patients with diffuse bone marrow involvement; however, concurrent multiparametric flow cytometry or next-generation sequencing can be used to overcome this problem.^9^

Our study aimed to review the radio-graphical findings in PET/CT at the time of diagnosis, together with the disease characteristics from biochemical results and bone marrow point of view, and to assess post treatment response from radiological, biochemical and bone marrow aspect.

## Methods

This retrospective cohort study was carried out at the Adult Hematology Division at King Abdulaziz Medical City, Riyadh, Saudi Arabia. Our retrospective study was granted the Institutional Review Board of the King Abdullah International Medical Research Centre (KAIMRC) approval (RC19/301/R). Our study patients were those who were newly diagnosed with MM between January 2010-December 2018, who usually referred to hematology service from other specialties such as Internal Medicine, Nephrology, or Orthopedic surgery. The aim of this study is to identify the correlation between PET scan pre and post treatment, as we evaluated the clinical and radiological response.

The inclusion criteria of this study were patients who received either chemotherapy/novel agents with or without hematopoietic stem cell transplantation (HSCT), with baseline and follow up end of treatment imaging. Patients without baseline study or follow up PET CT were excluded. Data were collected using tumor board discussion template of multiple myeloma patients. PET CT findings pre- and post-treatment were analyzed by our institutional nuclear medicine radiologists. Raw data were processed in accordance with the best practices for raw data management to identify any inaccuracies or incompleteness in advance of the statistical analysis.

### Statistical analysis

All interval variables were checked and summarized in terms of maximum and minimum values. These values were then checked and compared against the nominal ones, and the variables with implausible were flagged. All variables were summarized and reported for the study using descriptive statistics. Interval variables were summarized and reported in terms of mean and standard deviation. Categorical variables were summarized and reported in terms of frequency distribution and were compared using chi-square or Fisher’s exact test. All demographic and clinical variables were summarized for all cohorts. The max standardized uptake value (SUVmax) among clinical disease response were summarized using box plot. The Kruskal-wallis one way analysis of variance test was used to compare SUVmax values among clinical disease response. The bar diagrams were used to summarize patient responses during second and third PET/CT scan at the end of study. Statistical analysis was carried out in SAS software version 9.4. Positron emission tomography images were reviewed for foci of high metabolic activity as compared to the background. Corresponding CT findings were examined for morphologic characterization of the lesions. High metabolic activity associated with CT lesions was documented as myelomatous. The lesions were counted in each region. For semi-quantitative analysis of the FDG uptake, region of interest was drawn on the most prominent lesion on PET images, and the SUVmax was measured. A note was also made of the hypermetabolic lesions in soft tissue and other organs. Based on the visual and quantitative data we categorized the patients as follow: Study was recorded as positive scan when hypermetabolic lesions were seen and as a negative scan when tracer distribution was normal. Extent of the disease based on the number of lesions (Limited extent when the number of lesions were 5 or less, moderate extent when 6-10 lesions were noted and extensive when more than 10 lesions were noted in the whole body), degree of metabolic activity (mild when SUVmax was 5 or less and severe when the SUVmax was more than 5), and presence or absence of extramedullary lesions.

## Results

The mean age of our patients was 55.9 years old. Disease characteristics are shown in Table 1. Immunoglobulin (Ig) G Kappa multiple myeloma were most commonly diagnosed with percentage of 37.8%, the rest of the subtypes are outlined in Table 1. Most of the patients had International Staging System (ISS) staging of 2 and Durie-Salmon of 3a, and almost 81.08% had standard risk. The laboratory values and types of abnormal cytogenetics that were found in bone marrow biopsy are summarized in Table 2.

**Table 1:** - Demographics and disease characteristics.

Parameters	n (%)
Age, mean±SD	55.9±9.15
***Disease subtype***	
IgG Kappa	14 (37.8)
Free Kappa light chain	9 (24.3)
IgG Lambda	4 (10.8)
IgA Lambda	3 (8.1)
Free Lambda light chain	2 (5.4)
IgA Kappa	2 (5.4)
Non-secretory	2 (5.4)
IgA lambda/poems	1 (2.7)
***ISS staging***	
1	5 (13.51)
2	18 (48.64)
3	14 (37.84)
***Durie salmon staging***	
3a	27 (73.0)
2a	4 (10.8)
3b	4 (10.8)
1a	1 (2.7)
1b	1 (2.7)
***Risk stratification***	
Standard	30 (81.08)
High risk	7 (18.92)

Ig: immunoglobulin, ISS: International Staging System, SD: standard deviation

**Table 2:** - Laboratory data and cytogenetics abnormalities.

Monoclonal protein variables and cytogenetic characteristics	n (%)
IgG, median (Q1,Q3)	14.6 (7.42, 25.70)
IgM, median (Q1,Q3)	0.3 (0.17, 0.60)
IgA, median (Q1,Q3)	1.7 (0.60, 2.95)
***Immunofixation, n (%)***	
Present	27 (73)
Absent	10 (27)
Kappa, median (Q1,Q3)	20.2 (2.97, 155.00)
Lambda, median (Q1,Q3)	9.5 (1.74, 25.60)
Kappa/Lambda ratio, median (Q1,Q3)	3.2 (0.68, 30.61)
LDH, mean±SD	209.2 (115.87)
Beta micro globulin, Median (Q1,Q3)	4.3 (2.11, 7.39)
***Cytogenetics, n (%)***	
Normal	26 (56.5)
Abnormal	20 (43.5)
***Type of abnormal cytogenetic, n (%)***	
No abnormality	26 (56.5)
Hyperdiploidy	10 (21.7)
Hypodiploidy	4 (08.7)
t(11;14)	1 (02.2)
Deletion of chromosome 17	1 (02.2)
Monosomy 13	1 (02.2)
Rearrangement Igh gene and ccnd1 gene	1 (02.2)

Ig: immunoglobulin, ISS: International Staging System, SD: standard deviation

Initial FDG PET/CT was performed in 37 patients. A follow up scan before autologous stem cell transplant (ASCT) or starting maintenance therapy was available in 37 while end of therapy scan was done in 20 patients. Bone lesions were seen in all except one patient on the initial scan. However, 12 patients had less than 5 lesions and the rest had 5 lesions or more. Lytic lesions with soft tissue involvement were noted in 12 patients. Lymph nodes and other soft tissue organs involvement such as liver, spleen, oropharynx and pancreas were noted in 9 patients. At the end of therapy PET/CT scan, no bony lesions were shown in 12 patients, less than 5 lesions were seen in 6 patients, and 5 or more bony lesions were seen in 2 patients. In regards the lymph nodes and soft tissue involvement at the end of therapy PET/CT scan this was seen in 2 patients Table 3.

**Table 3:** - Distribution of positron emission tomography/computed tomography (PET/CT) findings on different body involved areas (N=39).

Parameters	First PET/CT(n=37)	Second PET/CT(n=37)	Third PET/CT(n=20)
PET/CT done	37 (100)	37 (100)	20 (100)
***Therapy status***			
Post chemotherapy	37 (100)	37 (100)	20.0 (100)
***Number of lesions***			
<5	12 (32.4)	19 (51.6)	6.0(30.0)
≥5 and <10	5 (13.5)	6 (18.2)	0
≥10	19 (51.4)	4 (12.1)	2.0(10.0)
No bony lesion	1 (2.7)	8 (21.6)	12.0(60.0)
Skull, mean±SD	2.6±6.02	0.2±1.04	0.3±1.52)
Spine, mean±SD	6.5±6.95	2.3±3.00	1.8±2.72)
Upper limbs including scapula, mean±SD	5.1±8.51	2.2±6.97	0.9±2.45
Lower limbs, mean±SD	4.8±9.14	1.5±4.52	0.8±2.31
Pelvic bones, mean±SD	7.1±8.10	2.4±2.66	1.9±2.42
Ribs + sternum, mean±SD	6.3±5.50	2.16±2.58	1.9±2.59
***Patterns of bone lesion***			
Lytic	19 (51.4)	17 (46.0)	12 (60.0)
lytic lesion with soft tissue	12 (32.4)	4 (10.8)	1 (5.0)
Lytic and sclerotic	3 (8.1)	12 (32.4)	5 (25.0)
Sclerotic	1 (2.7)	2 (5.4)	1 (5.0)
Diffuse osteopenia	1 (2.7)	0.0	
No bone lesion	1 (2.7)	2 (5.4)	1 (5.0)
Lymph nodes (presents)	2 (5.4)	2 (5.4)	1 (5.0)
***Organs involved***			
No organ involved	30 (81.1)	0	19 (95.0)
Liver	2 (5.4)	2 (5.4)	1 (5.0)
Spleen	2 (5.4)	30 (81.1)	0
Soft tissue	1(2.7)	5 (13.5)	0
Nasopharynx and lungs	1 (2.7)	0	0
Pancreas	1 (2.7)	0	0

Values are presented as number and percentages (%) unless otherwise stated. SD: standard deviation

A number of 20 patients achieved clinical complete response, 50% of them had 10 or more bony lesions at end of therapy study, while 12 of them had radiological complete response, and 8 patients had partial, stable disease or progressive disease.

**Figure 1:**
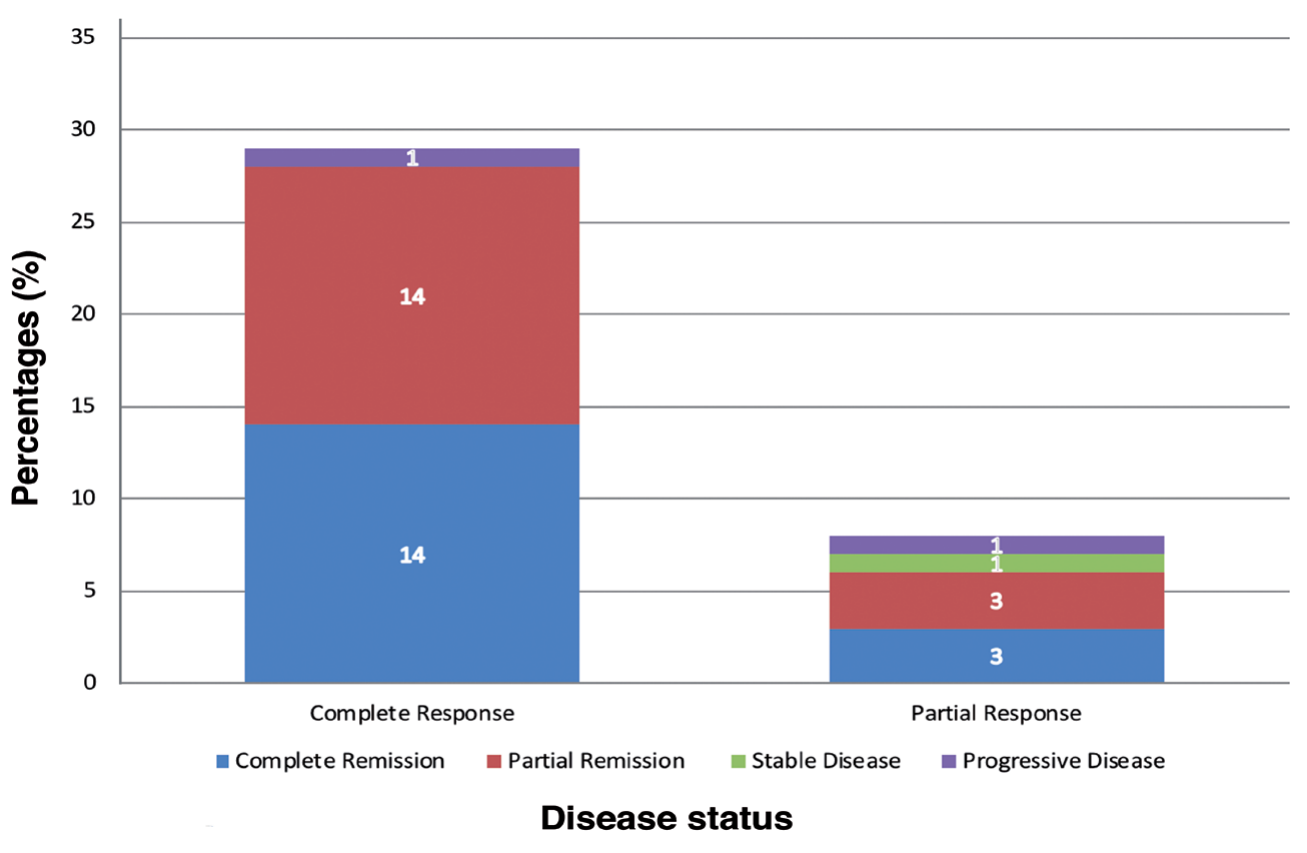
- Distribution of patient responses during second positron emission tomography computed tomography (PET CT) scan and end of study. The x-axis represents the clinical responses at the end of the study and y-axis represents no-of patient’s responses during second PET scan.

In regard to follow up PET/CT scans that were done prior to proceeding with ASCT, clinical correlation was made with PET/CT findings. We found that 17 patients were in complete radiological remission by PET/CT, however only 14 patients of this group showed complete clinical remission Figure 1. Furthermore, one patient who had a clinical complete response showed a progressive disease in the second PET/CT, which could be due to delay in appearance of bone disease in relation to therapeutic status.

**Figure 2:**
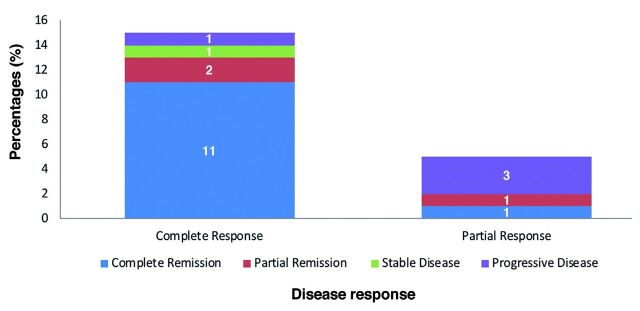
- Distribution of patient responses during third positron emission tomography computed tomography (PET CT) scan and end of study. The x-axis represents the clinical responses at the end of the study and y-axis represents no-of patient’s responses during third PET scan.

For the end of therapy assessment of both clinical and radiological findings, we found that out of the 15 patients who had a clinical complete remission, 11 were in radiologic remission, 2 had partial remission, 1 had stable disease, and one patient had progressive disease Figure 2.

**Figure 3:**
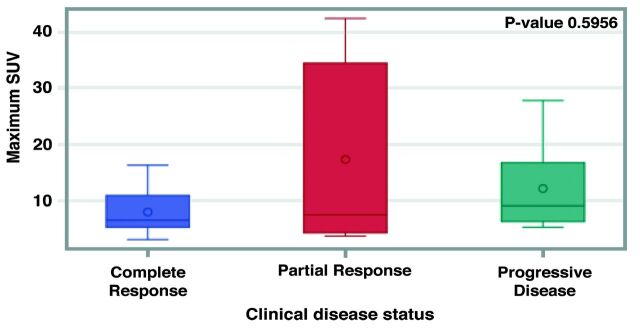
- Comparison of Maximum SUV values with clinical disease status at the end of the study.

A comparison of SUVmax values shown on PET/CT scan at the end of the study with the clinical disease status shown as complete remission, partial remission and progressive disease is shown in Figure 3.

## Discussion

Multiple myeloma is a plasma cell dyscrasia characterized by multi-organ involvement including osteoclastic activation leading to osteolytic bony lesions observed in 80% of MM patients.^10^ It is one of the most frequent presentations therefore prompt evaluation by imaging techniques is the corner stone of diagnosis of bone involvement in MM patients. In terms of diagnosis, skeletal survey is less sensitive than PET/CT although it was used to be the gold standard modality for evaluation of bony lesions.^11^ Nowadays, PET/CT images are obtained routinely initially at the diagnosis, then to follow up and assess the disease response since it is considered as the best modality.^2,12,13^ Based on systemic review that was carried out on PET/CT which showed a sensitivity of 80-90% and a specificity of 80-100% for PET/CT in detecting osteolytic lesions, this was strongly emphasized in our study as a 97.3% of patients had osteolytic bone lesions that were detected in initial PET/CT, with highest SUV uptake detected in pelvic bones, followed by the spine. Fifty one percent of these lesions were lytic in nature, lymph nodes were present in 5.4%, and liver and splenic involvement was observed in 5.4% for both.^14^

Serial scanning in MM has incremental and prognostic value as documented in previous published reports.^9,14,15^ Those patients who show a decline in the metabolic activity and the number of lesions often achieve a complete response. This is in agreement to the study carried out by Cavo et al^9^ where they stated that suppression of avidity of FDG-PET/CT was associated with significant increase of overall survival.

Positron emission tomography/computed tomography is known to be a useful tool to evaluate response to treatment, however, there is still no set criteria for scoring a lesion on post therapy scans to label as the disease resolution or probably a residual lesion. This may be difficult with bony lesions where activity on PET/CT at lesions due to healing process may mimic increase in the metabolic activity. Moreover, bone marrow activation due to chemotherapy may also be challenging to exclude or report the disease.

The comparison of radiological response measured as SUV value shown on PET/CT scan with the clinical disease status at the end of the study was consistent with the complete clinical responders as shown of SUVmax less than 10. As expected with partial clinical remission group this has shown the highest SUV activity where it exceeded 30. In the progressive disease group, the SUV activity was less than 20 but this can be explained by biochemical and cellular disease progression with probably a delay in the bone disease involvement.

To assess the disease severity by measuring the metabolic activity as SUV max and disease burden by assigning the frequency of bone/bone marrow lesions, we did not find a statistically significant correlation between the response versus (vs.) metabolic activity or the number of bone/bone marrow lesions. However, a trend was noted where patients with SUVmax of >5 were more likely to achieve a complete or partial response than those patients with SUV max <5. However, we noted that there is no clear correlation between SUV max value and clinical response. For our patients, 85% of patients who had complete response reported an SUV max >5. In contrast, all patients who had progressive disease had an SUV max >5. Noting that there is no clear cut off of metabolic activity to serve as a surrogate for patient’s outcome.

Our study was limited by the absence of clear criteria for defining disease response in PET/CT. Many independent studies advocated for standardization of interpreting findings of PET/CT. Further prospective analysis would better clarify these issues, and subsequently establish a defined criterion.

### Study limitation

Sample size can not be magnifies simply due to the fact that not all our MM patients had PET scan pre and post treatment therefore quite few patients had to be excluded from the study. The other important limitation factor was implementing PET scan in the staging and end of treatment evaluation was a routine only the last few years therefore many patients were staged with skeletal survey which is not an inclusion criteria for our cohort.

Positron emission tomography scan response criteria for MM patients and adopting the International Working Myeloma Group criteria of clinical response and lastly implementing upfront PET scan as a radiological staging of the disease. Furthermore, as low dose density CT Scan is a very useful way in assessing MM patients’ response to treatment, we need to do more head to head studies with PET scan statistics with PET scan.

In conclusion, although we did not find a statistically significant correlation between the response vs. metabolic activity and the number of bone/bone marrow lesions, however, our study was limited by the absence of clear criteria for defining disease response in PET/CT in MM patients. We believe further prospective analysis with larger sample size will elaborate more on this important correlation.

## References

[1] World Health Organization. Global Cancer Observatory: Cancer Today. [Updated 2020; 2021 March 5] Available from: https://gco.iarc.fr/today

[2] Hillengass J , Moulopoulos LA , Delorme S , Koutoulidis V , Mosebach J , Hielscher T , et al. Whole-body computed tomography versus conventional skeletal survey in patients with multiple myeloma: a study of the International Myeloma Working Group. Blood Cancer J 2017; 7: e599. 28841211 10.1038/bcj.2017.78 PMC5596388

[3] Rajkumar SV , Dimopoulos MA , Palumbo A , Blade J , Merlini G , Mateos MV , et al. International Myeloma Working Group updated criteria for the diagnosis of multiple myeloma. Lancet Oncol 2014; 15: e538–e548. 25439696 10.1016/S1470-2045(14)70442-5

[4] Moreau P , Attal M , Caillot D , Macro M , Karlin L , Garderet L , et al. Prospective evaluation of magnetic resonance imaging and [(18)F]Fluorodeoxyglucose positron emission tomography-computed tomography at diagnosis and before maintenance therapy in symptomatic patients with multiple myeloma included in the IFM/DFCI 2009 trial: results of the IMAJEM study. J Clin Oncol 2017; 35: 2911–2918. 28686535 10.1200/JCO.2017.72.2975 PMC5578392

[5] Bannas P , Kröger N , Adam G , Derlin T . [Modern imaging techniques in patients with multiple myeloma]. RoFo 2013; 185: 26–33. 23196838 10.1055/s-0032-1325405

[6] Sachpekidis C , Mai EK , Goldschmidt H , Hillengass J , Hose D , Pan L , et al. (18)F-FDG dynamic PET/CT in patients with multiple myeloma: patterns of tracer uptake and correlation with bone marrow plasma cell infiltration rate. Clin Nucl Med 2015; 40: e300–e307. 25783508 10.1097/RLU.0000000000000773

[7] Zamagni E , Tacchetti P , Cavo M . Imaging in multiple myeloma: How? When? Blood 2019; 133: 644–651. 30587527 10.1182/blood-2018-08-825356

[8] Baffour FI , Glazebrook KN , Kumar SK , Broski SM . Role of imaging in multiple myeloma. Am J Hematol 2020; 95: 966–977. 32350883 10.1002/ajh.25846

[9] Cavo M , Terpos E , Nanni C , Moreau P , Lentzsch S , Zweegman S , et al. Role of (18)F-FDG PET/CT in the diagnosis and management of multiple myeloma and other plasma cell disorders: a consensus statement by the International Myeloma Working Group. Lancet Oncol 2017; 18: e206–e217. 28368259 10.1016/S1470-2045(17)30189-4

[10] Haghighat S . The Role of 18F-FDG-PET/CT Scan in the management of multiple myeloma. APJCC 2020; 5: 119–123.

[11] Rajkumar SV . Multiple myeloma: 2018 update on diagnosis, risk stratification, and management. Am J Hematol 2018; 93: 981–1114. 30400719 10.1002/ajh.25117 PMC6223128

[12] Kosmala A , Bley T , Petritsch B . Imaging of multiple myeloma. RoFo 2019; 191: 805–816. 31185511 10.1055/a-0864-2084

[13] Rasch S , Lund T , Asmussen JT , Lerberg Nielsen A , Faebo Larsen R , Østerheden Andersen M , et al. Multiple myeloma associated bone Disease. Cancers (Basel) 2020; 12: 2113. 32751464 10.3390/cancers12082113 PMC7465468

[14] Dammacco F , Rubini G , Ferrari C , Vacca A , Racanelli V . ¹⁸F-FDG PET/CT: a review of diagnostic and prognostic features in multiple myeloma and related disorders. Clin Exp Med 2015; 15: 1–18. 25218739 10.1007/s10238-014-0308-3

[15] Ziogas DC , Dimopoulos MA , Kastritis E . Prognostic factors for multiple myeloma in the era of novel therapies. Expert Rev Hematol 2018; 11: 863–879. 30334460 10.1080/17474086.2018.1537776

